# Century-scale Methylome Stability in a Recently Diverged *Arabidopsis thaliana* Lineage

**DOI:** 10.1371/journal.pgen.1004920

**Published:** 2015-01-08

**Authors:** Jörg Hagmann, Claude Becker, Jonas Müller, Oliver Stegle, Rhonda C. Meyer, George Wang, Korbinian Schneeberger, Joffrey Fitz, Thomas Altmann, Joy Bergelson, Karsten Borgwardt, Detlef Weigel

**Affiliations:** 1 Department of Molecular Biology, Max Planck Institute for Developmental Biology, Tübingen, Germany; 2 Machine Learning and Computational Biology Research Group, Max Planck Institute for Developmental Biology and Max Planck Institute for Intelligent Systems, Tübingen, Germany; 3 European Molecular Biology Laboratory, European Bioinformatics Institute, Wellcome Trust Genome Campus, Hinxton, Cambridge, United Kingdom; 4 The Leibniz Institute of Plant Genetics and Crop Plant Research, Gatersleben, Germany; 5 Department of Plant Developmental Biology, Max Planck Institute for Plant Breeding Research, Cologne, Germany; 6 Department of Ecology and Evolution, University of Chicago, Chicago, Illinois, United States of America; 7 Center for Bioinformatics (ZBIT), Eberhard Karls Universität Tübingen, Tübingen, Germany; National Institute of Genetics, Japan

## Abstract

There has been much excitement about the possibility that exposure to specific environments can induce an ecological memory in the form of whole-sale, genome-wide epigenetic changes that are maintained over many generations. In the model plant *Arabidopsis thaliana*, numerous heritable DNA methylation differences have been identified in greenhouse-grown isogenic lines, but it remains unknown how natural, highly variable environments affect the rate and spectrum of such changes. Here we present detailed methylome analyses in a geographically dispersed *A. thaliana* population that constitutes a collection of near-isogenic lines, diverged for at least a century from a common ancestor. Methylome variation largely reflected genetic distance, and was in many aspects similar to that of lines raised in uniform conditions. Thus, even when plants are grown in varying and diverse natural sites, genome-wide epigenetic variation accumulates mostly in a clock-like manner, and epigenetic divergence thus parallels the pattern of genome-wide DNA sequence divergence.

## Author Summary

It continues to be hotly debated to what extent environmentally induced epigenetic change is stably inherited and thereby contributes to short-term adaptation. It has been shown before that natural *Arabidopsis thaliana* lines differ substantially in their methylation profiles. How much of this is independent of genetic changes remains, however, unclear, especially given that there is very little conservation of methylation between species, simply because the methylated sequences themselves, mostly repeats, are not conserved over millions of years. On the other hand, there is no doubt that artificially induced epialleles can contribute to phenotypic variation. To investigate whether epigenetic differentiation, at least in the short term, proceeds very differently from genetic variation, and whether genome-wide epigenetic fingerprints can be used to uncover local adaptation, we have taken advantage of a near-clonal North American *A. thaliana* population that has diverged under natural conditions for at least a century. We found that both patterns and rates of methylome variation were in many aspects similar to those of lines grown in stable environments, which suggests that environment-induced changes are only minor contributors to durable genome-wide heritable epigenetic variation.

## Introduction

Differences in DNA methylation and other epigenetic marks between individuals can be due to genetic variation, stochastic events or environmental factors. Epigenetic marks such as DNA methylation are dynamic; they can be turned over during mitosis and meiosis or altered by chromatin remodeling or upon gene silencing caused by RNA-directed DNA methylation (RdDM). Moreover, changes in DNA sequence or structure caused by, for instance, transposable element (TE) insertion, can induce secondary epigenetic effects at the concerned locus [1], [2], or, via processes such as RdDM, even at distant loci [3]–[5]. The high degree of sequence variation, including insertions/deletions (indels), copy number variants (CNVs) and rearrangements among natural accessions in *A. thaliana* provides ample opportunities for linked epigenetic variation, and the genomes of *A. thaliana* accessions from around the globe are rife with differentially methylated regions (DMRs) [6]–[10], but it remains unclear how many of these cannot be explained by closely linked genetic mutations and thus are pure epimutations [11] that occur in the absence of any genetic differences.

The seemingly spontaneous occurrence of heritable DNA methylation differences has been documented for wild-type *A. thaliana* isogenic lines grown for several years in a stable greenhouse environment [12], [13]. Truly spontaneous switches in methylation state are most likely the consequence of incorrect replication or erroneous establishment of the methylation pattern during DNA replication [14]–[16]. A potential amplifier of stochastic noise is the complex and diverse population of small RNAs that are at the core of RdDM [17] and that serve as epigenetic memory between generations. The exact composition of small RNAs at silenced loci can vary considerably between individuals [13], and stochastic inter-individual variation has been invoked to explain differences in remethylation, either after development-dependent or induced demethylation of the genome [18], [19]. Such epigenetic variants can contribute to phenotypic variation within species, and epigenetic variation in otherwise isogenic individuals has been shown to affect ecologically relevant phenotypes in *A. thaliana* [20]–[22].

In addition to these spontaneous epigenetic changes, the environment can induce demethylation or de novo methylation in plants, for example after pathogen attack [23]. Recently, it has been proposed that repeated exposure to specific environmental conditions can lead to epigenetic differences that can also be transmitted across generations, constituting a form of ecological memory [24]–[27]. The responsiveness of the epigenome to external stimuli and its putative memory effect have moved it also into the focus of attention for epidemiological and chronic disease studies in animals [28], [29]. How the rate of trans-generational reversion among induced epivariants with phenotypic effects compares to the strength of natural selection, which in turn determines whether natural selection can affect the population frequency of epivariants, is largely unknown [30]–[33].

To assess whether a variable and fluctuating environment is likely to have long-lasting effects in the absence of large-scale genetic variation, we have analyzed a lineage of recently diverged *A. thaliana* accessions collected across North America. Using a new technique for the identification of differential methylation, we found that in a population of thirteen accessions originating from eight different locations and diverged for more than one hundred generations, only 3% of the genome had undergone a change in methylation state. Notably, epimutations at the DNA methylation level did not accumulate at higher rates in the wild as they did in a benign greenhouse environment. Using genetic mutations as a timer, we demonstrate that accumulation of methylation differences was non-linear, corroborating our previous hypothesis that shifts in methylation states are generally only partially stable, and that reversions to the initial state are frequent [12], [34]. Many methylation variants that segregated in the natural North American lineage could also be detected in the greenhouse-grown population, indicating that similar forces determined spontaneous methylation variation, independently of environment and genetic background. Population structure could be inferred from differences in methylation states, and the pairwise degree of methylation polymorphism was linked to the degree of genetic distance. Together, these results suggest that the environment makes only a small contribution to durable, trans-generationally inherited epigenetic variation at the whole-genome scale.

## Results

### Characterization of the near-isogenic HPG1 lineage from North America

**Figure 1:**
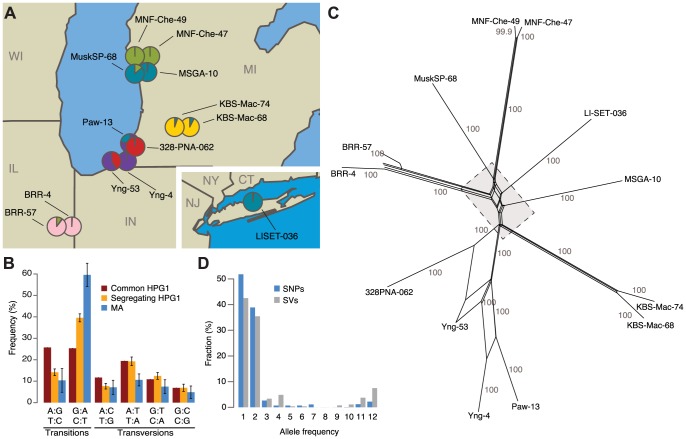
Identification of North American accessions that belong to a genetically homogeneous population. (A) Sampling locations of the 13 haplogroup-1 (HPG1) strains analyzed in this study. Pie charts indicate population structure inferred from segregating SNP data; SNP: single nucleotide polymorphism. Data were analyzed using STRUCTURE [66], with *K* = 6. CT = Connecticut, IL = Illinois, IN = Indiana, MI = Michigan, NJ = New Jersey, NY = New York, WI = Wisconsin. (B) Single-nucleotide mutation spectrum. Bars represent the accession average, error bars indicate 95% confidence intervals. (C) Phylogenetic network of HPG1 accessions based on segregating SNPs and structural variants (SVs) with SplitsTree v.4.12.3 [68]. Numbers indicate bootstrap confidence values (10,000 iterations). Dashed line delimits close-up in S6 Fig. (D) Allele frequencies of SNPs and SVs.

Previous studies of isogenic mutation accumulation (MA) lines raised in uniform greenhouse conditions identified many apparently spontaneously occurring pure epimutations [12], [13]. To determine whether variable and fluctuating environments in the absence of large-scale genetic variation substantially alter the genome-wide DNA methylation landscape over the long term, we analyzed a lineage of recently diverged *A. thaliana* accessions collected across North America. Different from the native range of the species in Eurasia, where nearly isogenic individuals are generally only found at single sites, about half of all North American individuals appear to be identical when genotyped at 139 genome-wide markers [35]. We selected 13 individuals of this lineage, called haplogroup-1 (HPG1), from locations in Michigan, Illinois and on Long Island, including pairs from four sites (Fig. 1A, S1 Table). Seeds of the accessions had been originally collected between 2002 and 2006 during the spring season, from plants at the end of their life cycle. Because rapid flowering in the greenhouse was dependent on an extended cold treatment, or vernalization, we conclude that the parental plants had germinated in autumn of the previous year and overwintered as rosettes. Climate data from the nearest respective weather station confirmed that precipitation and temperature regimes had varied considerably between sites in the growing season preceding collection (S1-S2 Fig.).

Whole-genome sequencing of pools of eight to ten siblings from each accession identified a shared set of 670,979 single nucleotide polymorphisms (SNPs) and 170,998 structural variants (SVs) relative to the Col-0 reference genome, which were then used to build a HPG1 pseudo reference genome (SOM: Genome analysis of HPG1 individuals; S2-S3 Table; S3 Fig.). Only 1,354 SNPs and 521 SVs segregated in this population (S4 Table, S4-S5 Fig.), confirming that the 13 strains were indeed closely related. Segregating SNPs were noticeably more strongly biased towards GC→AT transitions than shared SNPs, especially in TEs, although the bias was not as extreme as in the greenhouse-grown MA lines (Fig. 1B) [36]. A phylogenetic network and STRUCTURE analysis based on the segregating polymorphisms reflected the geographic origin of the accessions (Fig. 1A, C; S6 Fig.). Three of the pairs of accessions from the same site were closely related, and were responsible for many alleles with a frequency of 2 in the sampled population (Fig. 1D). If the spontaneous genetic mutation rate is similar to that seen in the greenhouse [36], the HPG1 accessions would be 15 to 384 generations separated from each other. With a generation time of one year, their most recent common ancestor would have lived about two centuries ago, which is consistent with *A. thaliana* having been introduced to North America during colonization by European settlers [37]. This is also in line with the fact that in several US herbarium collections, *A. thaliana* specimens from the mid-19^th^ century can be found, among these specimens from the Eastern Seaboard and the Upper Midwest. We conclude that the HPG1 accessions constitute a near-isogenic population that should be ideal for the study of heritable epigenetic variants that arise in the absence of large-scale genetic change under natural growth conditions. Because we observed only a weak positive correlation between genetic distance and phenotypic differences in the greenhouse (S7 Fig.), we also infer that life history differences on their own should have little effect on the epigenetic landscape.

### Spectrum and frequency of single-site DNA methylation polymorphisms

To assess the long-term heritable fraction of DNA methylation polymorphisms in the HPG1 lineage, we grew plants under controlled conditions for two generations after collection at the natural sites, before performing whole methylome bisulfite sequencing on two pools of 8–10 individuals per accession (S5 Table). We sequenced pools to reduce inter-individual methylation variation and fluctuations in methylation rate caused by stochastic coverage or read sampling bias. After mapping reads to the HPG1 pseudo reference genome, we first investigated epigenetic variation at the single-cytosine level. There were 535,483 unique differentially methylated positions (DMPs), with an average of 147,975 DMPs between any pair of accessions (SD  =  23,745); thus, 86% of methylated cytosines accessible to our analyses were stably methylated across all HPG1 accessions. The vast majority of variable sites (97%) were detected in the CG context (CG-DMPs). As we have discussed previously [12], this can be largely attributed to the lower average CHG and CHH methylation rates at individual sites compared to CG methylation, whereby differences in methylation rates are smaller and statistical tests of differential methylation fail more often for CHG and CHH sites.. Additionally, stable silencing-associated methylation of repeats and TEs, elements rich in CHG and CHH sites, may contribute to this pattern. That only about 2% of all covered cytosines were differentially methylated in the relatively uniform HPG1 population contrasted with a previous epigenomic study, in which most cytosines in the genome were found to be differentially methylated in 140 genetically divergent accessions [10]. Fewer than 10% of all cytosines in the genome were never methylated across these 140 accessions, although most methylation events were confined to single strains (S9 Table of ref. [10]). To make our data more comparable to this other study [10], we identified DMPs of each HPG1 accession against the Col-0 reference genome. On average we found 383,237 DMPs per accession, affecting a total of 1,046,892 unique sites. We estimated that we would have detected 3.6 million DMPs, if we had sequenced 140 accessions from the HPG1 lineage (see Materials and Methods; S8 Fig.). The considerably larger number of DMPs in the 140 accessions [10] is likely due both to different methodology and to the higher degree of genetic variation between the analyzed accessions. For example, Schmitz and colleagues [10] did not directly test for differential methylation at individual sites nor did they apply multiple testing correction, which might contribute to the high number of CHH-DMPs reported in that study.

**Figure 2:**
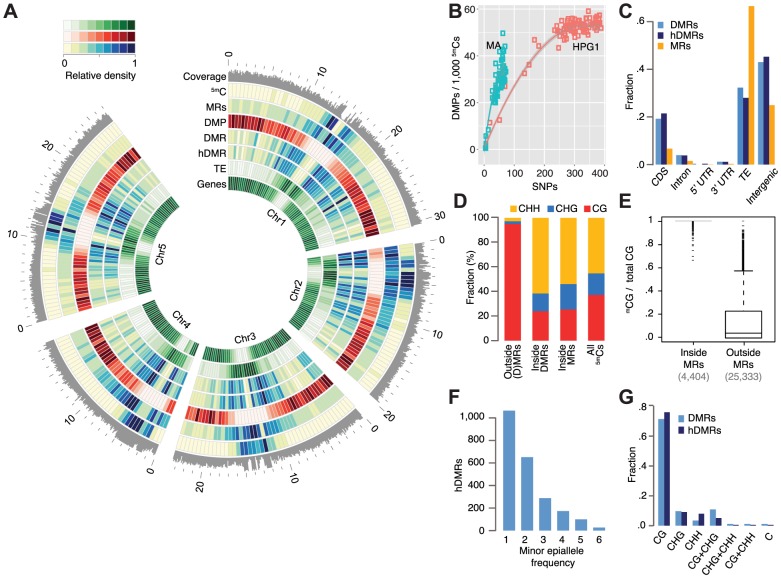
Epigenetic variation in a nearly isogenic population. (A) Genome-wide features: average coverage in 100 kb windows, the remainder in 500 kb windows. Outside coordinates in Mb. (B) Number of DMPs in relation to number of SNPs in pairwise comparisons. Data of mutation accumulation (MA) lines are based on single individuals, haplogroup1 (HPG1) data on pools of 8–10 individuals; each data point represents an independent comparison of two lines. DMPs in each pairwise contrast were scaled to the number of methylated sites compared. (C) Annotation of cytosines in MRs and hDMRs. (hD)MR sequences were assigned to only one annotation in the following order: CDS> intron. UTR> transposon> intergenic. (D) Sequence context of methylated positions relative to MRs and DMRs. (E) Fraction of ^5m^CGs among all CG sites for each gene and transposable element, with at least 5 CGs. (F) Minor epiallele frequencies of 2,304 hDMRs that could be split into only two groups and for which at least four strains showed statistically significant differential methylation. Strains not tested statistically significant for a particular hDMR were not considered for this plot. (G) DMRs and hDMRs according to sequence contexts in which significant methylation differences were found. ‘C’ denotes (h)DMRs in all three contexts. Abbreviations: 5mC: methylated position, CDS: coding sequence, DMP: differentially methylated position, DMR: differentially methylated region, hDMR: highly differentially methylated region, HPG1: haplogroup-1 lines, MA: mutation accumulation lines, MRs: methylated regions, SNP: single nucleotide polymorphism, TE: transposable element, UTR: untranslated region.

Using the geographic outlier LISET-036 as a reference strain, we found that 61% of CG-DMPs as well as 36% of the small number of CHG- and CHH-DMPs were present in at least two independent accessions (S9A Fig.), many of them shared between accessions from the same site. As is typical for *A. thaliana* [38], most methylated positions clustered around the centromere and localized to TEs and intergenic regions (Fig. 2A; S9B Fig.). In contrast, differential methylation in the CG context was over-represented on chromosome arms, localizing predominantly to coding sequences (Fig. 2A; S9B Fig.), similar to what we had previously observed in the greenhouse-grown MA lines [12].

We asked whether DMPs had accumulated more quickly in natural environments than in the greenhouse, using DNA mutations in the HPG1 and MA populations as a molecular clock (SOM: Estimating DMP accumulation rates). Our null hypothesis was that a variable and highly fluctuating natural environment increases the rate of heritable methylation changes. In contrast to this expectation, DMPs appear to have accumulated in sub-linear fashion in both the HPG1 and MA populations [12] (Fig. 2B) – with similar trends for DMPs in all three contexts – and the number of DMPs did not increase more rapidly in the HPG1 than in the MA lines. The steeper initial increase relative to SNP differences as well as the broader distribution of MA line differences relative to HPG1 differences were most likely the result of having compared individual plants in the MA experiment [12], rather than pools of siblings, as in the HPG1 experiment. The effect of pooling individuals, as shown by simulation (S10 Fig.), and a potentially higher genetic mutation rate in the wild than in the greenhouse, for example because of increased stress [39], could lead to a slight underestimation of the true HPG1 epimutation rate, but it remains unlikely that it greatly exceeds the one of the MA lines (SOM: Estimating DMP accumulation rates).

### Differentially methylated regions in the HPG1 lineage

Because it is unclear whether variation at individual methylated cytosines has any consequences in plants, we next focused on differentially methylated regions (DMRs) in the HPG1 population. A limitation of previous plant methylome studies using short read sequencing has been that these relied on integration over methylated or single differentially methylated sites, or on the analysis of fixed sliding windows along the genome to identify DMRs. What appears intuitively to be more appropriate is to first identify regions that are methylated in individual strains (SOM: Differentially methylated regions) [40], and to test only these for differential methylation. We therefore adapted a Hidden Markov Model (HMM), which had been developed for segmentation of animal methylation data [41], to the more complex DNA methylation patterns in plants. We identified on average 32,529 methylated regions (MRs) per strain (median length 122 bp), with the unified set across all strains covering almost a quarter of the HPG1 reference genome, 22.6 Mb (Fig. 2A, C; S11A Fig.; S6 Table). MRs overlapping with coding regions were over-represented in genes responsible for basic cellular processes (p-value <<0.001), in agreement with gene body methylation being a hallmark of constitutively expressed genes [42]. Only 1% of ^m^CHH and 2% of ^m^CHG positions were outside of methylated regions (Fig. 2D), consistent with the dense CHH and CHG methylation found in repeats and silenced TEs [38]. Compared to ^m^CGs within methylated regions,^ m^CGs in unmethylated space localized almost exclusively to genes (94%), were spaced much farther apart, and were separated by many more unmethylated loci (Fig. 2E; S11B-C Fig.). This explains why sparsely methylated genes were under-represented in HMM-determined methylated regions, even though gene body methylation accounts for a large fraction of methylated CG sites. The accuracy of our MR detection method was well supported by independent methods (SOM: Validation of methylated regions).

Using the unified set of MRs, we tested all pairs of accessions for differential methylation, identifying 4,821 DMRs with an average length of 159 bp (S12 Fig.; S11A Fig.; S7 Table). Of the total methylated genome space, only 3% were identified as being differentially methylated, indicating that the heritable methylation patterns had remained largely stable in this set of geographically dispersed accessions. Indeed, 91% of genic and 98% of the TE sequence space were devoid of DMRs. Of the DMRs, 3,199 were classified as highly differentially methylated (hDMRs; S8 Table), i.e. they had a more than three-fold change in methylation rate and were longer than 50 bp. The DMR allele frequency spectrum was similar to that of variably methylated single sites (Fig. 2F). Most DMRs and hDMRs showed statistically significant methylation variation in only one cytosine context, often CG (Fig. 2G), even though DMRs were dominated by CHG and CHH methylation (Fig. 2D, S13 Fig.). Different from individual sites (DMPs), the densities for DMRs and hDMRs were highest in centromeric and pericentromeric regions, and overlapped more often with TEs than with genes (Fig. 2A, C). Relative to all methylated regions, genic regions were two-fold overrepresented in the genome sequence covered by DMRs, and three-fold in hDMRs (Fig. 2C). Currently, we do not know whether this simply reflects the greater power of detecting differential methylation at the typically more highly methylated CG sites compared to CHG or CHH sites, or whether this reflects actual biology.

### Methylation variation and transcriptome changes

DNA methylation in gene bodies has been proposed to exclude H2A.Z deposition and thereby stabilize gene expression levels [42]. We therefore asked what impact differential methylation had on transcriptional activity. We identified 269 differentially expressed genes across all possible pairwise combinations (S9-S10 Table), most of which were found in more than one comparison. When we clustered accessions by differentially expressed genes, closely related pairs were placed together (S14 Fig.). We identified 28 differentially expressed genes that overlapped with an hDMR either in their coding or 1 kb upstream region, but the relationship between methylation and expression was variable (S11 Table). By visual examination, we found not more than five instances of demethylation that were associated with increased expression; examples are shown in S15 Fig.

### Comparison of epimutations in natural and greenhouse-grown lineages

**Figure 3:**
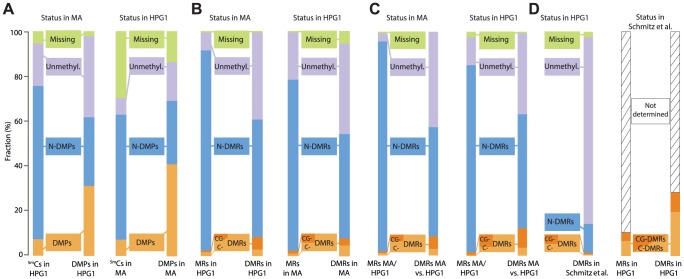
Overlapping epigenetic variation in independent populations. (A) Comparison of methylated positions (^5m^Cs) and differentially methylated positions (DMPs) identified in pairwise comparisons of mutation accumulation (MA) [12] and haplogroup-1 (HPG1). Distinction in different sequence contexts has been omitted since almost all DMPs (>97%) are in CG context. Left: sites in HPG1 strains and their status in the MA data; right: sites in the MA strains and their status in the HPG1 data. N-DMPs: non-differentially methylated positions. (B) Comparison of methylated regions (MRs) and differentially methylated regions (DMRs) identified in pairwise comparisons of HPG1 and MA lines. Dark and light orange subsets of DMRs distinguish regions with differential methylation occurring exclusively in CG context (CG-DMRs) or in any additional or alternative context(s) (C-DMRs). Left: regions in HPG1 strains and their status in the MA data; right: regions in the MA strains and their status in the HPG1 data. (C) MRs and DMRs identified in comparison between one randomly chosen MA line (30–39) and one randomly chosen HPG1 line (MuskSP-68), and their overlap with within-population DMRs. (D) Comparison of HPG1 DMRs with CG-DMRs (dark orange) from ref [10] and C-DMRs (light orange) identified in 140 natural *A. thaliana* accessions. Because methylated regions were not reported in ref. [10], the overlap of DMRs with the space not covered by DMRs could not be assessed. N-DMRs: non-differentially methylated regions.

With the caveat that there are uncertainties about the genetic mutation rate in the wild, and therefore how the number of SNPs relates to the number of generations since the last common ancestor, there was no evidence for faster accumulation of variably methylated sites in the HPG1 population, nor for very different epimutation rates among HPG1 lines (Fig. 2B). Importantly, the overlap of differential methylation between the two populations was much greater than expected by chance: the probability of a random ^m^C site in the MA population of being variably methylated in the HPG1 population was only 7%, but it was 41% among sites that were also variably methylated in the MA population – a six-fold enrichment (four-fold enrichment in the reciprocal comparison; Fig. 3A). In other words, almost half of the DMPs in the MA lines were also polymorphic in the HPG1 lines, and almost a third of HPG1 DMPs were also variably methylated in the MA population. These shared DMPs were more heavily biased towards the chromosome arms and towards genic sequences than population-specific epimutations (S16A-S16B Fig.). Conversely, DMPs unique to one population were more likely to be unmethylated throughout the other population when compared to random methylated sites (Fig. 3A), as one might expect for sites that sporadically gain methylation.

DMPs unique to the HPG1 lineage appeared to be less frequent in the pericentromere compared to MA- line-specific DMPs (S16A Fig.), which was also reflected in an apparently higher epimutation frequency in the MA lines for these regions (S16B Fig.). We therefore investigated whether the annotation spectrum differed between these two classes of differentially methylated sites. Even though MA-specific DMPs were more often found in TEs compared to HPG1-specific DMPs, this bias was also observed for all cytosines accessible to our methylome analyses (S16C Fig.), and can therefore be explained by a more accurate read mapping and better TE annotation in the Col-0 reference compared to the HPG1 pseudo-reference genome. Indeed, except for chromosome 4, the average sequencing depth in the pericentromere was higher in the MA lines (S16B Fig.).

DMPs distinguishing MA lines that were separated from each other by only a few generations were more frequently variably methylated in the HPG1 lineage than DMPs identified between distant MA lines (S17 Fig.). We interpret this observation as an indication of privileged sites that are more labile and therefore more likely to have already changed in status after a small number of generations.

We used the methods implemented for the HPG1 population to detect DMRs also in the MA strains. Similar to variable single positions, or DMPs, the overlap between 2,523 DMRs of the MA lines that we could map to the HPG1 methylated genome space with the 4,821 DMRs of the HPG1 accessions was greater than expected and highly significant (Ζ-score  = 32.9; 100,000 permutations). HPG1 DMRs were four-fold more likely to coincide with MA DMRs than with a random methylated region from this set (Fig. 3B). We observed similar degrees of overlap independently of sequence context. Shared DMRs between both lineages were, in contrast to shared DMPs, not biased towards genic regions (S18 Fig.). Differentially methylated regions in the HPG1 lineage, however, overlapped with genic sequences more often than MA DMRs (S18 Fig.), which might again be explained by the different efficiencies in mapping to repetitive sequences and TEs (S16B Fig.).

We next wanted to know how this short-term variation compared to methylation variation across much deeper splits. To this end, we identified variably methylated regions between a randomly chosen MA line and a randomly chosen HPG1 line; these DMRs, which differentiate distantly related accessions, were also enriched in each of the two sets of within-population DMRs (MA or HPG1) (Fig. 3C). Finally, we compared DMRs found in the HPG1 population to DMRs that had been identified with a different method among 140 natural accessions from the global range of the species [10] (Fig. 3D). Although only 9,994, less than one fifth, of the variable regions from the global accessions were covered by methylated regions in the HPG1 strains, the overlap of DMRs was highly significant (Ζ-score  = 19.8; 100,000 permutations). Together, the high recurrence of differentially methylated sites and regions from different datasets points to the same loci being inherently biased towards undergoing changes in DNA methylation independently of genetic background and growth environment.

To explore potential sources of such lability, we compared variation in the HPG1 lines to that caused by mutations in various components of epigenetic silencing pathways [43]. Almost all variable sites and regions in CG-methylated parts of the HPG1 genome were hypomethylated in mutants deficient in DNA methylation maintenance, most notably in the *met1* single and the *vim123* triple mutants (S19 Fig.). This is consistent with polymorphic methylation arising primarily because of errors in the maintenance of symmetrical CG methylation during DNA replication. Hypermethylated sites in the *rdd* triple mutant, which shows impaired demethylation, were also found slightly more often within variably methylated regions of all contexts (S19D Fig.).

### Heritability and genetic linkage of epigenetic variation

**Figure 4:**
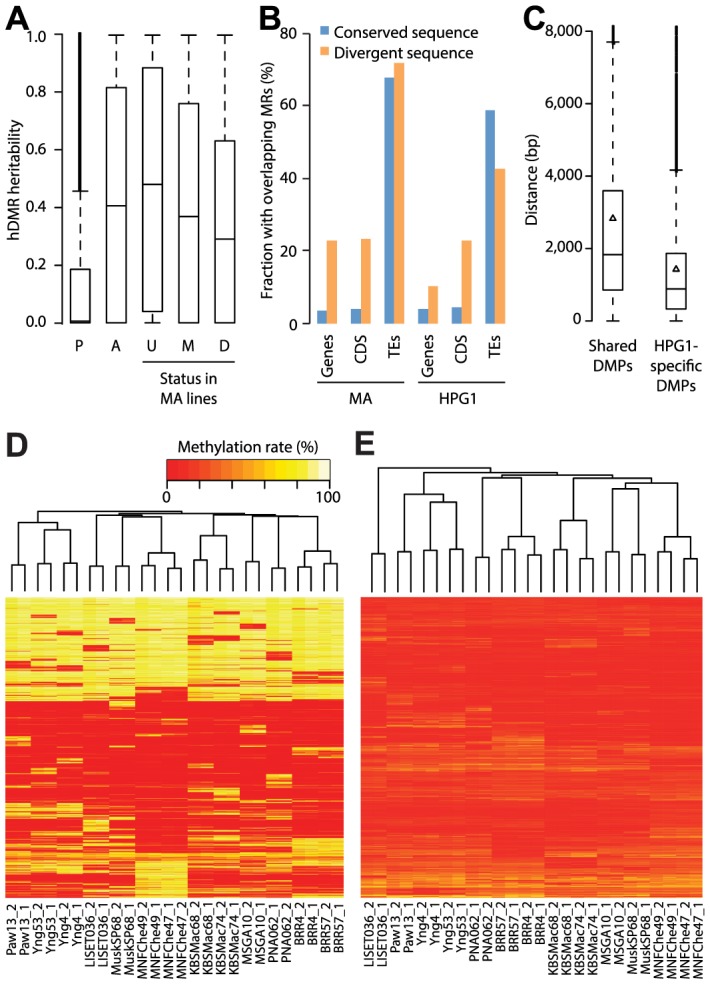
Genetic effects on epigenetic variation. (A) Heritability values based on genome-wide genetic differentiation for all highly differentially methylated regions (hDMRs), hDMRs with randomly permuted methylation rates and subsets of hDMRs depending on their overlap with methylated and differentially methylated regions of the mutation accumulation (MA) population, respectively. P: Permuted (2,945 hDMRs); A: All (2,945); U: Unmethylated in MA (1,310); M: Methylated in MA (1,243); D: DMR in MA (392). (B) Correlation between structural variants (SVs) and probability of overlap with methylated regions (MRs). Divergent sequences are insertions of at least 20 bp relative to the other population. This analysis is based on 3,256 SVs overlapping with genes, 641 with coding sequences (CDS) and 4,020 with transposable elements (TEs) (S12 Table). (C) Distances between common SVs of at least 20 bp and the closest differentially methylated position (DMP), depending on whether it is shared between the mutation accumulation (MA) and haplogroup-1 (HPG1) populations. Triangles represent the mean. (D) Hierarchical clustering of HPG1 strains based on methylation rates at 50,000 CG-DMPs. (E) Hierarchical clustering of HPG1 strains based on average methylation rates of 2,829 hDMRs with full information across all strains. Methylation rates per region were calculated as the average methylation rate of each methylated cytosine in that region.

To quantify how many methylation differences were co-segregating with genome-wide genetic changes at both linked and unlinked sites, we estimated heritability for each highly differentially methylated region by applying a linear mixed model-based method. We used segregating sequence variants with complete information as genotypic data and average methylation rates of hDMRs with complete information as phenotypes. The median heritability of all hDMRs was 0.41 (mean 0.44), which means that genetic variance across the entire genome contributed less than half of methylation variance (Fig. 4A). hDMRs in the HPG1 strains that were not methylated in the greenhouse-grown MA lines had a higher median heritability, 0.48, than HPG1 hDMRs also found among MA DMRs (0.29), which held true for all sequence contexts (Fig. 4A; S20 Fig.). Regions of highly differential methylation found only in the HPG1 population, especially those in unmethylated regions of the MA lines, were thus more likely to be linked to whole-genome sequence variation than hDMRs found in both populations. For 19% of all hDMRs (21% CG-hDMRs, 14% CHG-hDMRs, 7% CHH-hDMRs), the whole-genome genotype explained more than 90% of their methylation differences (with a standard error of at most 0.1). Of these hDMRs, half had a heritability of greater than 0.99. That 6.7% of the sequence space of these heritable hDMRs still overlapped with MA DMRs (versus 9.4% for the less heritable hDMRs) was in agreement with the hypothesis that there are regions that vary highly in their methylation status independently of genetic background.

To identify genetic variants that potentially directly cause methylation changes in their local genomic neighborhood, we focused on variably methylated regions that were within 1 kb of segregating SNPs or indels. Of 191 such DMRs, only three showed a systematic correlation with nearby sequence polymorphisms. We noticed, however, that coding regions with structural variants larger than 20 bp that distinguished the MA and HPG1 populations were more likely to be methylated in both lineages than non-polymorphic coding regions (Fig. 4B). Consequently, DMPs unique to the HPG1 lines were on average closer to insertions or deletions than DMPs shared between the HPG1 and MA populations (Fig. 4C).

Lastly, we asked whether the genome-wide methylation pattern reflected genetic relatedness, i.e., population structure. Hierarchical clustering by methylation rates of variable sites and regions grouped strains by sampling location (Fig. 4D, E). This result was largely independent of the sequence or the annotation context of these loci, and not seen with sites that our statistical tests had identified as stably methylated (S21 Fig.). That variably methylated regions grouped the accessions similar to DMPs, albeit with less confidence (shorter branch lengths; S21 Fig.), suggested that our DMR calling algorithm was conservative. Methylation data thus paralleled similarity between accessions at the genetic level, in agreement with the interpretation that methylation differences primarily reflect the number of generations since the last common ancestor.

## Discussion

We have tested the hypothesis that accumulation of epigenetic variation under natural conditions proceeds over the short term in a very different manner than the clock-like behavior of genetic variation [24]–[27]. To this end, we have taken advantage of a unique natural experiment, the *A. thaliana* HPG1 lineage, which has likely diverged for at least a century throughout North America. Our analyses have revealed little evidence for broad-scale and durable epigenetic differentiation that might have been induced by the variable and fluctuating environmental conditions experienced by the HPG1 accessions since they separated from each other. While the exact conditions these plants have been subjected to since their separation from a common ancestor remain unknown, the time scale and diversity of geographic provenance are strong indicators of the variability of the environment between the different sampling sites, supported by temperature and precipitation data from nearby weather monitoring stations. The general analytical framework enabled by the HPG1 lineage – nearly isogenic lines grown for more than a century under variable and fluctuating conditions – could not have been achieved in a controlled greenhouse experiment.

Studies of epiRIL populations have shown that pure epialleles can be stably transmitted across several generations [5], [19], but how often this is the case for environmentally induced epigenetic changes has been heavily debated [33], [44]–[46]. The recent excitement about the transmission of induced epigenetic variants comes from such variants having been proposed to be more often adaptive than random genetic mutations [24]–[26]. Contrary to the expectations discussed above, we found that epimutation rates under natural growth conditions at different sites did not differ substantially from those observed in a controlled greenhouse environment, with polymorphisms accumulating sub-linearly in both situations, apparently because of frequent reversions. Note that we grew the HPG1 plants under controlled conditions for two generations after sampling at the natural site, to reduce the range of epigenetic variation to the long-term heritable fraction. Given that the environment can induce acute methylation changes [23], [47], it is likely that we would have observed greater epigenetic variation, if we had sampled field-grown individuals directly. However, most of such variation induced during ontogeny does not appear to be heritable, as we did not find evidence for it after two extra generations in the greenhouse. Additional studies that directly compare plants grown outdoors to their progeny grown in a stable and controlled environment will help to further clarify this issue.

We found that positions of differential methylation in the HPG1 population are more likely to overlap with DMPs detected between closely related MA lines than between more distantly related MA lines. This observation supports the hypothesis that there are different classes of polymorphic sites. One of these includes ‘high lability’ sites that are independent of the genetic background, that change with a high epimutation rate, and that are therefore more likely to appear in each population. Another class of DMPs comprises more stable sites that gain or lose methylation more slowly and that therefore are less likely to be shared between different populations.

Differences between accessions in terms of DNA methylation recapitulated their genetic relatedness, further corroborating our hypothesis that heritable epigenetic variants arise predominantly as a function of time rather than as a consequence of rapid local adaptation. Epigenetic divergence thus does not become uncoupled from genetic divergence when plants grow in varying environments, nor does the rate of epimutation noticeably increase. A minor fraction of heritable epigenetic variants may be related to habitat, which could be responsible for LISET-036 being epigenetically a slight outlier (Fig. 4E), even though it is not any more genetically diverged from the most recent common ancestor of HPG1 than other lines. Such local epigenetic footprints could also explain fluctuations in epimutation frequency between the MA and HPG1 lineages. Subtle adaptive changes at a limited number of loci would go unnoticed in the present analysis of genome-wide patterns and can therefore not be excluded. However, on a genome-wide scale there was little indication of adaptive change: neither were LISET-036 specific regions of differential methylation in and near genes enriched for GO terms with an obvious connection to environmental adaptation, nor were there overlapping differentially expressed genes (S22 Fig., SOM: Analysis of LISET-036 specific hDMRs). In combination with the general lack of correlation between differential methylation and changes in gene expression, our findings suggest that epigenetic changes in nature are mostly neutral, and thus comparable to genetic mutations. We point out that an annual species such as *A. thaliana* might be differently disposed to record environmental signals in its epigenome compared to more long-lived species. From an evolutionary perspective, in perennial species the advantage of epigenetically mediated local adaptation to changing conditions could be more pronounced, and future studies are warranted to address this question.

Because of the near-isogenic background of the HPG1 accessions, we were also able to gauge how much of epigenetic variation is either caused by, or stably co-segregates with genetic differences. HPG1-specific highly differentially methylated regions were more often linked to genotype variation than regions that were variably methylated in both the HPG1 and MA populations. This suggests that heritable hDMRs can, to a certain extent, be considered facilitated epigenetic changes [11].

Both differentially methylated regions and positions are over-represented in genes, but TEs and intergenic regions contain many variable regions and only very few variable single sites. Altogether our data indicate that both variably and constitutively methylated positions in genes are typically separated by many unmethylated sites and that a large fraction of these is therefore not classified as being (differentially) methylated. Variability of DNA methylation in plant genes thus mainly affects single, sparsely distributed cytosines, the biological relevance of which remains unclear.

Our comparisons between MA laboratory strains and natural HPG1 accessions have revealed that loci of variable methylation overlapped much more between the two groups than expected by chance, despite these populations having experienced very different environments that also differ greatly in their uniformity, and despite completely different genetic backgrounds. The observation that changes at many sites and loci are independent of the genetic background and geographic provenance suggests that spontaneous switches in methylation predominantly reflect intrinsic properties of the DNA methylation and gene silencing machinery, with the CG maintenance system seemingly being the most error-prone. Our most important finding is probably that DNA methylation is highly stable across dozens, if not hundreds of generations of growth in natural habitats; 97% of the total methylated genome space was not contained in a DMR. The stark contrast to published data, which describes more than 90% of cytosines in the genome as variably methylated in a set of 140 divergent natural accessions [10], can be explained both by the low amount of genetic divergence among the HPG1 accessions and by methodological differences. For future studies, we recommend the application of non-permissive statistical tests in the analysis of differential methylation. The overall stability of methylation presented here is in accordance with the high similarity of methylation in evolutionarily conserved gene sequences [48]. It contrasts, however, with our recent report showing that over longer evolutionary distances that separate species in the same genus or closely related genera, there is very little conservation of global DNA methylation, simply because the sequences that are typically methylated are much more evolutionarily fluid than non-methylated sites [47]. In summary, we propose that the stability of DNA methylation first and foremost depends on the stability of the underlying genetic sequence and that heritable polymorphisms that arise in response to specific growth conditions appear to be much less frequent than those that arise spontaneously. These conclusions are of importance when considering epimutations as a potential evolutionary force.

## Materials and Methods

### Plant growth and material

Accessions [35] were collected in the field at locations indicated in S1 Table. Seeds had been bulked in the Bergelson lab at the University of Chicago before starting the experiment. Plants were then grown at the Max Planck Institute in Tübingen on soil in long-day conditions (23°C, 16 h light, 8 h dark) after seeds had been stratified at 4°C for 6 days in short-day conditions (8 h light, 16 h dark). We grew one plant of each accession under these conditions; seeds of that parental plant were then used for all experiments. Eight plants of the same accession were grown per pot in a randomized setup. All accessions used in this paper have been added to the 1001 Genomes project (http://1001genomes.org) and have been submitted to the stock center.

### Nucleic acid extraction

DNA was extracted from rosette leaves pooled from eight to ten individual adult plants. Plant material was flash-frozen in liquid nitrogen and ground in a mortar. The ground tissue was resuspended in Nuclei Extraction Buffer (10 mM Tris-HCl pH 9.5, 10 mM EDTA, 100 mM KCl, 0.5 M sucrose, 0.1 mM spermine, 0.4 mM spermidine, 0.1% β-mercaptoethanol). After cell lysis in nuclei extraction buffer containing 10% Triton-X-100, nuclei were pelleted by centrifugation at 2000 *g* for 120 s. Genomic DNA was extracted using the Qiagen Plant DNeasy kit (Qiagen GmbH, Hilden, Germany). Total RNA was extracted from rosette leaves pooled from eight to ten individual adult plants using the Qiagen Plant RNeasy Kit (Qiagen GmbH, Hilden, Germany). Residual DNA was eliminated by DNaseI (Thermo Fisher Scientific, Waltham, MA, USA) treatment.

### Library preparation

DNA libraries for genomic and bisulfite sequencing were generated as described previously [12]. Libraries for RNA sequencing were prepared from 1 µg of total RNA using the TruSeq RNA sample prep kit from Illumina (Illumina) according to the manufacturer's protocol.

### Sequencing

All sequencing was performed on an Illumina GAII instrument. Genomic and bisulfite-converted libraries were sequenced with 2×101 bp paired-end reads. For bisulfite sequencing, conventional *A. thaliana* DNA genomic libraries were analyzed in control lanes. Transcriptome libraries were sequenced with 101 bp single end reads. Four libraries with different indexing adapters were pooled in one lane; no control lane was used. For image analysis and base calling, we used the Illumina OLB software version 1.8.

### Processing of genomic reads

The SHORE pipeline v0.9.0 [49] was used to trim and quality-filter the reads. Reads with more than 2 (or 5) bases in the first 12 (or 25) positions with a base quality score of less than 4 were discarded. Reads were trimmed to the right-most occurrence of two adjacent bases with quality values equal to or greater than 5. Trimmed reads shorter than 40 bases and reads with more than 10% (of the read length) of ambiguous bases were discarded.

Reads were aligned against the *Arabidopsis thaliana* genome sequence version TAIR9 in iteration 1 and against updated “Haplogroup 1-like” genomes in further iterations. The mapping tool GenomeMapper v0.4.5s [50] was used, allowing for up to 10% mismatches and 7% single-base-pair gaps along the read length to achieve high coverage. All alignments with the least amount of mismatches for each read were reported. A paired-end correction method was applied to discard repetitive reads by comparing the distance between reads and their partner to the average distance between all read pairs. Reads with abnormal distances (differing by more than two standard deviations) were removed if there was at least one other alignment of this read in a concordant distance to its partner. The command line arguments used for SHORE are listed in S1 File.

### Genetic variant identification

Genetic variants were called in an iterative approach. In each step, SNPs and structural variants common to all strains were detected and incorporated into a new reference genome. The thus refined “HPG1-like” genomes served as the reference sequence in the subsequent iterations (S3 Fig.). We performed three iterations to call segregating variants and built two reference genomes to retrieve common polymorphisms. The steps performed in each iteration are described in the following paragraphs.

### SNP and small indel calling

Base counts on all positions were retrieved by SHORE v0.9.0 [49] and a score was assigned to each site and variant (SNP or small indel of up to 7% of read length) depending on different sequence and alignment-related features. Each feature was compared to three different empirical thresholds associated with three different penalties (40%, 20% and 5% reduction of the score, initial score: 40). They can be found in S13 Table.

For comparisons across lines, positions were accepted if at most one intermediate penalty on their score was applicable to at least one strain (score ≥32). In this case, the threshold for the other strains was lowered, accepting at most one high and two intermediate penalties (score ≥15). In this way, information from other strains was used to assess sites from the focal strain under the assumption of mostly conserved variation, allowing the analysis of additional sites.

Only sites sufficiently covered (≥5x) and with accepted base calls in at least half of all strains (≥7 out of 13) were processed further. Variable alleles with a frequency of 100% were classified as "common" and variants with a lower frequency as "segregating".

Additional SNPs were called using the targeted *de novo* assembly approach described below.

### Structural variant (SV) calling

Although a plethora of SV detection tools have been developed, the predicted variants show little overlap between each other on the same data sets. Furthermore, the false positive rate of many methods can be drastic [51]. Hence, rather than taking the intersection of the output from different tools, which would yield only a small number of SVs, we combined different tools and applied a stringent evaluation routine to identify as many true SVs as possible. Since SVs common to all strains should be incorporated into a new reference, only methods that identify SVs on a base pair level could be used. Currently, there are four different SV detection strategies (based on depth of coverage, paired-end mapping, split read alignments or short read assembly, respectively). Only tools based on split read alignments and assemblies are capable of pinpointing SV breakpoints down to the exact base pair. Programs that were used include Pindel v2.4t [52], DELLY v0.0.9 [53], SV-M v0.1 [54] and a custom local *de novo* assembly pipeline targeted towards sequencing gaps (described below). We reported deletions and insertions from all tools, and additionally inversions from Pindel. DELLY combines split read alignments with the identification of discordant paired-end mappings. Thus, our SV calling made use of three out of four currently available methodologies.

Reads for DELLY were mapped using BWA v0.6.2 [55] against the TAIR9 Col-0 reference genome to produce a BAM file as DELLY's input format.

The arguments for the command line calls of all tools are listed in S1 File.

### Targeted *de novo* assembly

While using a re-sequencing strategy, there are regions without read coverage (“sequencing gaps”) because either the underlying sequence is being deleted in the newly sequenced strain, or highly divergent to the reference sequence, or present in the focal strain, but not represented in the read set. To access sequences in the first two classes, a local *de novo* assembly method was developed.

Insertion breakpoints or small deletions, however, can mostly not be detected by zero coverage due to reads ranging with a few base pairs into or beyond the structural variants. Therefore, we defined a “core read region” as the read sequence without the first and last 10 nucleotides. To be able to assemble the latter cases, the definition of “sequencing gaps” was extended from zero-covered regions to stretches not spanned by a single read's core region.

All reads aligned to the surrounding 100 nucleotides of such newly defined sequencing gaps as well as the unmappable reads from the re-sequencing approach together with their potential mapped partners constituted the assembly read set. Two assembly tools were used to generate contigs, SOAPdenovo2 v2.04 [56] and Velvet v1.2.0 [57] (command line arguments in S1 File). Contigs shorter than 200 bp were discarded. To map the remaining contigs of each assembler against the iteration-specific reference genome, their first and last 100 bp were aligned with GenomeMapper v0.4.5s [50], accepting a maximal edit distance of 10. If both contig ends mapped uniquely within 5,000 bp, the thus framed region on the reference was aligned to the contig using a global sequence alignment algorithm after Needleman-Wunsch (‘needle’ from the EMBOSS v6.3.1 package). In addition, non-mapping contigs were aligned with blastn (from the BLAST v2.2.23 package) [58] to yield even more variants.

All differences between contig and reference sequences were parsed (including SNPs, small indels and SVs) for each assembly tool. Only identical variants retrieved from both assemblers were selected.

### Generating and filtering consolidated variant set of each strain

For each strain, all variants from the SV tools and the *de novo* assemblies were consolidated (S3A Fig.) and positioned to consistent locations to be comparable using the tool Dindel v1.01 [59]. In the case of contradicting or overlapping variants, identical variants (having the same coordinates and length after re-positioning) predicted by a majority of tools were chosen and the rest discarded, or all were discarded if there was no majority.

Despite sequencing errors or cross-mapping artifacts of the re-sequencing approach, genomic regions covered by reads are generally trusted. Chances of long-range variations in the inner 50% of a mapped read's sequence (“inner core region” of a read) are assumed to be low, since gaps would deteriorate the alignment capability towards the ends of the read.

Therefore, we filtered out variants from the consolidated variant set spanning a genomic region already covered by at least one inner core region of a mapped read of the corresponding strain (S3A Fig.), assuming homozygosity throughout the genome. This “core read criterion” had to be fulfilled at each genomic position spanned by the variant.

### Using branched reference to validate variants

Variants passing the core read filter in all strains were classified as common variants and were incorporated into the reference sequence of the previous iteration, thus replacing the reference allele. Segregating variants, which could not be detected in all strains, were additionally built into the reference in separate “haplotype regions” (or “branches” of the reference sequence) to eventually be able to assess whether reads preferentially mapped to the reference or the alternative haplotype sequence (S3A Fig.). Linked variant haplotypes of a strain (distance between consecutive variants ≤107 bp, the maximal possible span of a read on the reference) as well as identical haplotype regions among strains were merged into one branch sequence.

For each strain, all reads were re-mapped to this new reference sequence yielding read counts at the variant site on each branch (r_b_) and at the corresponding site on the reference haplotype sequence (r_ref_) (S3A Fig.). Here, the read count of a site was defined as the number of inner core regions spanning the site. To increase certainty of variant calling and to rule out heterozygosity, the read count of the major allele was tested against a binomial distribution that assumed 95% allele frequency out of a total of r_b_+r_ref_ observations, i.e. sole presence of either the branch or the reference haplotype (if 100% had been assumed, it would not yield a distribution). The null hypothesis of homozygosity was rejected after *P* value correction by Storey's method [60] for *q* values below 0.05.

The same variant could be part of several different haplotypes and thus, could be included into different branch sequences. Reads supporting this variant would map at multiple locations in the reference. Therefore, we allowed all aligned rather than only unique reads to contribute to read counts and omitted the paired-end correction procedure.

### Final sets of common and segregating variants

We followed a similar “population-aware” approach to prefer commonalities among strains as was used for the SNP calling for labeling variants as being common or segregating. Here, variable sites with accumulated coverage over both branch and reference sequence of ≤3x were marked as “missing data”. If there was at least one haplotype in a strain with a *q* value above 0.05, it was assumed to be present in the population. If the test on the same haplotype failed in another strain, but the absolute read count of the haplotype sequence exceeded the alternative haplotype read count by ≥2-fold, then this haplotype was considered present in the corresponding strain as well.

We classified variants where at least 7 out of 13 strains did not show missing data as ‘common’ if the branched haplotype was present in all strains, as ‘not present’ if the reference haplotype was present in all strains, or into ‘segregating’ if there was support for both haplotypes.

To combine common variants identified by the described stepwise algorithm into potentially less evolutionary events, we aligned 200 bp around each variant of the last iteration's genome back to the TAIR9 Col-0 reference genome using a global alignment strategy (‘needle’ from the EMBOSS v6.3.1 package).

In total, we found 842,103 common and 2,017 segregating polymorphisms without removing linked loci compared to Col-0 after two iterations, to which the different tools contributed to different extent depending on the variant type (S3C Fig.).

### Methylome sequencing

Genomic and bisulfite sequencing were performed as described in ref. [12].

### Processing and alignment of bisulfite-treated reads

The procedure followed one described [12], except that we aligned reads against the HPG1-like as well as against the Col-0 reference genome sequences. Command line arguments for SHORE are listed in S1 File.

### Determination of methylated sites

We performed whole methylome bisulfite sequencing to an average depth of 18x per strand (S5 Table) on two pools consisting of 8-10 individuals per accession. We followed the same procedures as described [12] to retrieve statistically significantly methylated positions. Here, we restricted the set of analyzed positions to cytosine sites with a minimum coverage of 3 reads and sufficient quality score (Q25) in at least half of all strains (i.e. ≥7), that is, 21 million positions in total. Out of those, we identified 3.8 million methylated cytosines in at least one strain by applying a false discovery rate (FDR) threshold at 5%, and between 2,120,310 and 2,927,447 methylated sites per strain (S5 Table). False methylation rates retrieved from read mapping against the chloroplast sequence can be found in S5 Table. Using the HPG1 pseudo reference genome instead of the Col-0 reference genome increased the number of cytosines sufficiently covered for statistical analysis by 5% on average, and the number of positions called as methylated by 7% (S5 Table).

### Identification of differentially methylated positions (DMPs)

We performed the same methods as in ref [12] to obtain DMPs. First, cytosine positions were tested for statistical difference between both replicates of a sample using Fisher's exact test and a 5% FDR threshold. Because individual samples consisted of a pool of several plants, the number of DMPs between replicates was negligible (between 0 and 161). After excluding them, we applied Fisher's exact test on the 3.8 million cytosine sites methylated in at least one strain for all pairwise strain comparisons. Using the same *P* value correction scheme as in Becker *et al*., we identified 535,483 DMPs across all 13 strains.

### DMPs identified in dependence of the number of accessions

Using the model developed in ref [61], a beta prior distribution was estimated that determined the non-ancestral frequency for each variable site. We assumed the methylation state in Col-0 to be ancestral, which resulted in beta distribution parameters of a = 0.029 and b = 0.644, corresponding to a mean non-ancestral DMP frequency of 0.043 and a corresponding standard deviation of 0.157. These were then used to estimate the fraction of common DMPs that were expected to be found by sequencing a given number of methylomes. Based on the formula presented in supporting section 3 of ref [61], we estimated the total number of DMPs in the population:



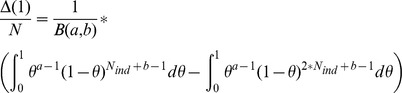



For N_ind_ = 13 (the number of accessions in this study) and Δ(1)  = 1,046,892 (the total number of DMPs versus the Col-0 reference), we estimated a total number of possible DMPs in the population of N = 59,770,415, which is close to the 43 million cytosines in the *A. thaliana* genome. Given such an estimate for N, the Δ function can be evaluated numerically to estimate the number of DMPs we would have detected had we analysed the same number of accessions as in ref [10] (S8 Fig.).

### Identification of methylated regions (MRs)

The value of an approach that defines methylated regions (MRs) before identifying differentially methylated regions (DMRs) has been demonstrated before with a Hidden Markov Model (HMM) method developed for the analysis of methylated-DNA-immunoprecipitation followed by array hybridization (MeDIP-chip) [40]. An HMM based on next-generation sequencing data was also applied to segment the maize genome, which is much more highly methylated than the *A. thaliana* genome, into hypo- and hypermethylated regions [62]. We modified the HMM implementation from Molaro and colleagues [41] based solely on within-genome variation in methylation rate. It assumes that the number of methylation-supporting reads at each cytosine follows a beta binomial distribution and that distributions over positions within and between methylated regions will differ from each other, providing a way to distinguish them. Thus, the model learns methylation rate distributions for both an unmethylated and a methylated state for each sequence context separately (CG, CHG and CHH) while simultaneously estimating transition probabilities between the two states from genome-wide data. On the trained model, the most probable path of the HMM along the genome is then used to define regions of high and low methylation. The method of Molaro and colleagues was designed for calling MRs in human samples, where the vast majority of methylated cytosines are in a CG context. In plants, however, one observes considerable methylation in all three contexts (CG, CHG and CHH), each with a different methylation rate distribution. Hence, we extended the HMM by learning the parameters of three different beta binomial distributions per state, one for each context. Additionally, in contrast to humans, only the minority of cytosines in the CG context is methylated, as are cytosines in the other contexts. Hence, methylation rates were inverted to find hypermethylated, rather than hypomethylated regions as in the original HMM implementation.

Apart from these changes and some final filtering steps (see below), we followed the same computational steps as described by Molaro and colleagues [41]: The describing parameters of the – in our case – six distributions (determining the emission probabilities) and the transition probabilities between states were iteratively trained (using the Baum-Welch algorithm) from methylation rates of all cytosines in the corresponding context throughout the genome. After each iteration, all cytosines were probabilistically classified into the most likely state via Posterior Decoding, given the trained model. After training of the HMM, i.e. after maximally 30 iterations or when convergence criteria were met, consecutive stretches of high methylation state were scored, in our case by the sum of all contained methylation rates. Next, *P* values were computed by testing the scores against an empirical distribution of scores obtained by random permutation of all cytosines throughout the genome. After FDR calculation, consecutive stretches in high state with an FDR <0.05 are defined as methylated regions (MRs).

The HMM was run on all genome-wide cytosines, independent of their coverage. Methylation rates were obtained using accumulated read counts from the strain replicates, resulting in one segmentation of the genome per strain. Gaps of at least 50 bp without a covered C position within a high methylation state automatically led to the end of the high methylation segment. Positions with a methylation rate below 10% at the start or end of highly methylated regions (until the first position with a rate larger than 10%), were assigned to the preceding or subsequent low methylation region, respectively.

### Identification of differentially methylated regions (DMRs)

The method to identify MRs yielded 13 different segmentations of the genome, one for each strain. We selected regions being in different or highly methylated states between strains and statistically tested them for differential methylation (including FDR calculation). To obtain epiallele frequencies, we clustered strains into groups based on their pairwise comparisons and statistically tested the groupings against each other. Regions that showed statistically significant methylation differences between at least two sets of strains were identified as DMRs. Finally, because of the sensitivity of the statistical test, we empirically filtered DMRs for strong signals and defined highly differentially methylated regions (hDMRs). All these steps are described in depth in the following.

### Selecting regions to test for differential methylation

We defined a breakpoint set containing the start and end coordinates of all predicted methylated regions. Each combination of coordinates in this set defined a segment to perform the test for differential methylation in all pairwise comparisons of the strains, if at least one strain was in a high methylation state throughout this whole segment (S12A Fig.). To also detect quantitative differences rather than solely presence/absence methylation, we also compared entirely methylated regions in more than one strain to each other.

Because of the sheer number of such regions, we applied the following greedy filter criteria: Regions were discarded from any pairwise comparison if less than 2 strains contained at least 10 cytosines covered by at least 3 reads each (accumulated over strain replicates) in this region (S12A Fig. (a)). Regions were discarded from any pairwise comparison if the reciprocal overlap of this region to at least one previously tested region was more than or equal to 70% (S12A Fig. (b)). This was done to prevent “similar” regions to be tested twice. Pairwise tests of a region were not performed if both strains were in low methylation state throughout the whole region (S12A Fig. (c)). Strains were excluded from pairwise comparisons in a region if the number of positions covered by at least 3 reads each was less than half of the maximum number of such positions of all strains in the same region (S12A Fig. (d)). This prevented comparing regions with unbalanced coverage to each other, e.g. a strain with 10 data points against another one with only 2.

These filters reduced the set of regions to test from ∼2.5 million to ∼230,000 per pairwise comparison.

### Testing regions for differential methylation between strains

We designed a statistical test for differential methylation between two strains for a given region. The test assumes that the number of methylated and unmethylated read counts per position along a region follows a beta binomial distribution – similar to the HMM in MR calling. More precisely, there are 3 distributions for each sequence context and for each strain. Using gradient-based numerical maximum likelihood optimization, we fitted the parameters for each beta binomial distribution on the available read count data of the region in the respective strain. This was done a) for each of the two strains separately (while taking strain replicates into account), resulting in (two times three) *strain-specific* beta binomial distributions, and b) for the read counts of both strains including their replicates together, resulting in (three) *common* beta binomial distributions. In this way, we obtained each distribution's mean µ and standard deviation σ. We selected only regions for potential DMRs, whose intervals [µ_1_ – 2σ_1_, µ_1_ + 2σ_1_] for strain 1 and [µ_2_ – 2σ_2_, µ_2_ + 2σ_2_] for strain 2 did not overlap.

To further corroborate statistical significance, we computed *P* values by calculating the ratio of the *strain-specific* and the *common* log likelihoods of the available read count data using the corresponding beta binomial distributions and by testing it against a chi-squared distribution (with 6 degrees of freedom). Let sample *S* have *N_Sc_* cytosines in context *c* in total and *C_Scp_* reads at position *p* in context *c*, from which *x_Scp_* are methylated, then we compute:



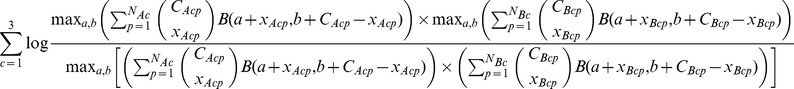



After correction for multiple testing using Storey's method [60], an FDR threshold of 0.01 defined statistically different methylated regions (DMRs) between two strains.

Additionally, this method allowed calling differential methylation in a region for each context separately by computing *P* values as described above without summing over the contexts (*c* = 1, 2 or 3). We termed resulting DMRs CG-DMRs if the methylation at only CG sites within this region was statistically significantly different, and similarly CHG-DMRs and CHH-DMRs.

### Grouping differentially methylated strains in each region

For 13 strains there are at maximum 78 pairwise comparisons per region. To summarize pairwise comparisons and obtain epiallele frequencies, we assigned strains into differentially methylated groups. To achieve such clustering, we constructed a graph for each region where strains were represented as vertices and connected to other strains by an edge if the region was identified as a DMR between them (S12B Fig.). We assume that strains within a group are then similarly methylated. The task is to find the smallest number of groups of vertices so that no two strains within a group are connected by an edge.

We set up a custom algorithm, which iteratively solves the “vertex coloring problem” for an increasing number of different colors, starting with two and quitting once all strains could be successfully assigned a color (S12B Fig.). In each iteration, strains were processed in descendent order of their degree (i.e. number of edges it is connected to). Each strain was assigned to all possible colors that did not invoke a collision. Subsequently, the algorithm continued recursively to assign the color of the next strain.

Each strain had 3 context-dependent means of its beta binomial distributions per region (termed *strain means* from now on). We roughly approximated each group's mean methylation values (*group means*) as the mean values of all *strain means* within a group. The *grouping diversity* describes the accumulated absolute differences between the *strain means* and their respective *group means* divided by the number of strains. As an example, consider S12B Fig. For simplicity, it only displays methylation rates for one out of three contexts. In the real data, the respective values were accumulated over all three contexts. The *group mean* for the blue strains in the example is (89+90+90+93+87)/5 = 89.8% and for the white strains 52%. The *grouping diversity* of the clustering shown here would be (from strains A to K): (|56–52|+|59–52|+|64–52|+|89–89.8|+|41–52|+|93–89.8|+|90–89.8|+|45–52|+|47–52|+|90–89.8|+|45–52|+|87–89.8|)/11 = 2.84.

If there was more than one possible grouping of the strains, we chose the one with the lowest *grouping diversity*. A strain with no edges (i.e. which is not statistically differentially methylated to any other strain) was assigned into the group to which the accumulated absolute difference between its *strain mean* and the *group mean* was lowest. In the example of S12B Fig., strain L is grouped to the blue strains because its mean methylation value (81%) is closer to the blue *group mean* (90%) than to the white one (52%).

This procedure summarized the ∼221,000 DMRs of all pairwise strain comparisons into 11,323 DMRs between groups of strains.

### Testing regions for differential methylation between groups of strains

Once grouped, the same statistical test as for differential methylation between two strains was used to test groups of strains. Beta binomial distributions were approximated using the read counts of all strains in a group as if they were replicate data. This procedure identified 10,645 groups of regions showing significantly different methylation. Because the method used for the selection of the regions to perform the differential test can result in overlapping regions, DMRs can still overlap each other. From sets of overlapping DMRs, the non-overlapping DMR(s) with the lowest ‘grouping diversity’ was (were) retained, resulting in 4,821 final DMRs. For the vast majority of DMRs (98%), strains were classified into two groups, i.e. there are only two epialleles.

### Identification of highly differentially methylated regions (hDMRs)

Our sensitive statistical test classified as differential some regions with low variance and only subtle methylation difference; we therefore defined as highly differentially methylated regions (hDMRs) with potentially greater biological relevance all DMRs that were longer than 50 bp and that showed a more-than-three-fold difference in methylation rate in at least one sequence context, when considering at least five cytosines of that context (S12 Fig.). In addition, the overall methylation rate of the DMR in the more highly methylated strain had to be greater than 20%. Of 3,909 size-filtered DMRs, 3,199 (80%) were classified as hDMRs (S8 Table). The grouping of hDMRs yielded similar epiallele frequencies as for the DMPs (54% with frequency larger than 1; Fig. 2F).

### Epigenetic variation in HPG1 lines and methylation-deficient mutants

The data from Stroud and colleagues [43] contain position-wise methylation rates for each sample. We defined a single site as methylated in wild type (WT) if both Col-0 samples Col_WA034L3 and Col_WB023L8 had a methylation rate of 10% or higher, and if at least one of them is more than 20% methylated. We declared a site in a mutant sample as having ‘lost’ methylation where the wild type was methylated and the mutant showed a methylation rate of less than 10%. In contrast, a ‘gained’ methylation site had less than 10% methylation in at least one of the WT samples and more than 20% methylation in the mutant. To assess if epigenetic variation in the HPG1 lines is enriched at sites affected by impaired methylation machinery, for each mutant, we constructed a set of positions, which were methylated in WT, covered in the mutant sample (i.e. present with a rate in the mutant sample file), and which were covered in the HPG1 and MA populations. A site was considered covered in a population when more than half of the strains showed a high quality and a more than 3-fold covered base call (see ‘Determination of methylated sites’ or [12]). For those positions and different subsets thereof, the fractions of sites with gained or lost methylation in the mutant compared to the wild type samples were plotted in S19 Fig.

### Heritability analysis of methylated regions

For each differentially methylated region, we considered a linear mixed model to estimate the proportion of variance that is attributable to genetic effects (heritability) and its standard error. The approach is similar to variance component models used in GWAS, e.g. refs. [63], [64]. Briefly, we considered the log average methylation rate of DMRs as phenotype and assessed the variance explained by genotype using a Kinship model constructed from all segregating genetic variants. We considered only DMRs and genetic polymorphisms that had no missing data in all accessions.

### Population structure analysis

We identified non-synonymous SNPs using SHOREmap_annotate [65] and excluded them from population structure analyses. We ran STRUCTURE v.2.3.4 [66] with *K* = 2 to *K* = 9 with a burn-in of 50,000 and 200,000 chains for 10 repetitions and determined the best *K* value using the Δ*K* method [67]. The phylogenetic network was generated using SplitsTree v.4.12.3 [68].

### Mapping to genomic elements

We used the TAIR10 annotation for genes, exons, introns and untranslated regions; transposon annotation was done according to [69]. Positions and regions were hierarchically assigned to annotated elements in the order CDS> intron> 5′ UTR> 3′ UTR> transposon> intergenic space. We defined as intergenic positions and regions those that were not annotated as either CDS, intron, UTR or transposon.

Positions were associated to the corresponding element when they were contained within the boundaries of that element. (D)MRs were associated to a class of element if they overlapped with that class of element; a (D)MR could only be associated to one class of element. When summing up basepairs of an element class covered by (D)MRs, the number of basepairs of a (D)MR overlapping with that class of element were considered. In that case the space covered by a (D)MR could be assigned to different classes of elements, while each basepair of the (D)MR could be assigned to only one class.

### Overlapping region analysis

We tested for significant overlap of DMRs using multovl version 1.2 (Campus Science Support Facilities GmbH (CSF), Vienna, Austria). We reduced the genome space to the basepair space covered by MRs identified in at least one HPG1 accession. DMRs were considered in the analysis if their start and end positions were contained within the MR space. DMRs that only partially overlapped with the MR space were trimmed to the overlapping part. Overlap between DMRs from different datasets was analyzed by running 100,000 permutations of both DMR sets within the MR basepair space. multovl commands are listed in S1 File.

### Processing and alignment of RNA-seq reads

Reads were processed in the same way as genomic reads, except that trimming was performed from both read ends. Filtered reads were then mapped to the TAIR9 version of the Arabidopsis thaliana (http://www.arabidopsis.org) genome using Tophat version 2.0.8 with Bowtie version 2.1.0 [70], [71]. Coverage search and microexon search were activated. The command lines for Tophat are listed in S1 File.

### Gene expression analysis

For quantification of gene expression we used Cufflinks version 2.0.2[72]. We ran a Reference Annotation Based Transcript assembly (RABT) using the TAIR10 gene annotation (ftp://ftp.arabidopsis.org/home/tair/Genes/TAIR10_genome_release/TAIR10_gff3/) supplied with the most recent transposable element annotation [69] Fragment bias correction, multi-read correction and upper quartile normalization were enabled; transcripts of each sample were merged using Cuffmerge version 2.0.2, with RABT enabled. For detection of differential gene expression we ran Cuffdiff version 2.0.2 on the merged transcripts; FDR was set to <0.05 and the minimum number of alignments per transcripts was 10. Fragment bias correction, multi-read correction and upper quartile normalization were enabled. The command lines for the Cufflinks pipeline are listed in S1 File. Analysis and graphical display of differential gene expression data was done using the cummeRbund package version 2.0.0 under R version 3.0.1.

### Data visualization

When not mentioned otherwise in the corresponding paragraph, graphical displays were generated using R version 3.0.1 (www.r-project.org). Circular display of genomic information in Fig. 2A was rendered using Circos version 0.63 [73].

### Phenotyping

Leaf area was determined using the automated IPK LemnaTec System and the IAP analysis pipeline [74]. Plants were grown in a controlled-environment growth-chamber in an alpha-lattice design with eight replicates and three blocks per replicate, taking into account the structural constraints of the LemnaTec system. Each block consisted of eight carriers, each carrying six plants of one line. Stratification for 2 days at 6°C was followed by cultivation at 20/18°C, 60/75% relative humidity in a 16/8 h day/night cycle. Plants were watered and imaged daily until 21 days after sowing (DAS). Adjusted means were calculated using REML in Genstat 14^th^ Edition, with genotype and time of germination as fixed effects, and replicate|block as random effects.

### Environmental variability

Local temperature and liquid precipitation data was calculated from National Climatic Data Center (NCDC) Global Summary of Day (GSOD) data. Collection locations were matched to the closest weather station with <5% missing data for five years prior to the collection date. Cumulative liquid precipitation was calculated each year starting from January 1.

### Data accessibility

The DNA and RNA sequencing data have been deposited at the European Nucleotide Archive under accession number PRJEB5287 and PRJEB5331. A GBrowse instance for DNA methylation and transcriptome data is available at http://gbrowse.weigelworld.org/fgb2/gbrowse/ath_methyl_haplotype1/. DNA methylation data, MR coordinates and genetic variant information have also been uploaded to the genome browser of the EPIC consortium (https://www.plant-epigenome.org/; https://genomevolution.org/wiki/index.php/EPIC-CoGe) and can be accessed at http://genomevolution.org/r/939v. The software of our methylation pipeline can be downloaded at http://sourceforge.net/projects/methpipeline.

## Supporting Information

S1 Fig Recent temperature histories of samples. Samples were matched to the closest weather stations (distance in km). Daily temperature range (dark bars) and means (white points) of 330 days prior to the collection date, such that late year data reflect temperature ranges of the previous year. Five-year temperature ranges (light bars) and means (white line) indicate longer-term temperature variability. Different collection dates and locations both contribute to different means and variance of recent temperatures experienced by samples.
(EPS)

S2 Fig Recent precipitation histories of samples. Cumulative (from January 1) liquid precipitation at the weather stations closest to each collection site (distance in km). Black lines show yearly histories for five years prior to collection, the thick black line indicates the cumulative history of the previous 330 days. Blue line shows the LOESS estimate of mean precipitation accumulation over a year.
(EPS)

S3 Fig Iterative re-alignment strategy and statistics. (A) Iterative re-alignment approach to evaluate predicted variants and to build a HPG1 pseudo-reference genome. (1) For each strain, variants called by diverse structural variant detection tools and a local *de novo* assembly pipeline were combined into a consolidated variant set. (2) Variants with core read coverage were filtered out, the remaining variants were classified into common and potentially segregating. Brown triangles symbolize insertions/deletions, the brown X represents a SNP. (3) All common variants were incorporated into the reference genome. All segregating variants were introduced in branches of the reference genome, which incorporated polymorphisms linked by less than 107 bp. (4) After mapping the reads against the branched reference, a binomial test was performed in each strain and for each variable site to call the allele, i.e., to determine whether there was statistical evidence for the presence of only one haplotype covering the variant's coordinates. Variants with the same non-reference allele call in all strains were considered as “common”; those with a reference call in at least one strain and a variant call in at least one other strain were classified as “segregating”. (5) All common variants from the previous step were incorporated into the new reference sequence, and a new iteration was started over from (1), or this new genome served as the HPG1 pseudo-reference genome after iteration 2. (B) Increase of detected variants and decrease of unsequenced genome space and unmappable reads by iterative read mapping. The legend on the right side denotes absolute values after iteration 2. The reference value (100%) derives from the mapping against the Columbia-0 genome sequence (TAIR9), and for common variants it is the number of variants leading to the genome of iteration 1. Thus, ∼842,000 common variants led to the genome of iteration 2, ∼864,000 to the genome of iteration 3. (C) Composition of common polymorphisms by variant type (top) and by detection tool (bottom). Variants found by more than one tool contributed to the count for all respective tools.
(EPS)

S4 Fig Distribution of genetic variants along the five chromosomes. Relative density of common variants in 100 kb sliding windows with a step size of 10 kb. SNP: single nucleotide polymorphism, SV: structural variant.
(EPS)

S5 Fig Annotation of genetic variants. Polymorphisms were hierarchically assigned to CDS> intron> 5′ UTR> 3′ UTR> transposon> intergenic. HPG1: haplogroup-1 lines, SNP: single nucleotide polymorphism, SV: structural variant.
(EPS)

S6 Fig Magnification of the central area of the phylogenetic network in Fig. 1c. Numbers indicate bootstrap confidence values (10,000 iterations).
(EPS)

S7 Fig Phenotypic analysis. (A) Leaf growth measured over time (Materials and Methods). Error bars represent 95% confidence intervals. On average, 36 plants were measured per accession. (B) Correlation of genetic distance, represented by number of SNPs per pairwise comparison, and difference in leaf area at 21 days after germination.
(EPS)

S8 Fig Number of DMPs in dependence of the number of sequenced accessions. (A) Fraction of all DMPs of different allele frequencies that can be detected by sequencing a given number of accessions. We assumed the methylation state in the Col-0 reference as the ancestral state. Vertical lines indicate the number of accessions in this study (13) and in ref [10] (140). f: relative derived allele frequency. (B). Estimate of the number of DMPs detected by sequencing a given number of HPG1 accessions. Vertical lines mark the number of accessions sequenced in this study and in ref [10].
(EPS)

S9 Fig Differentially methylated positions (DMPs). (A) Epiallele frequencies of DMPs for CG sites only (left), and comparison of all three sequence contexts (right). (B) Annotation of invariantly (N-DMPs) and differentially (DMPs) methylated sites. Cytosines were hierarchically assigned to CDS> intron> 5′ UTR> 3′ UTR> transposon> intergenic.
(EPS)

S10 Fig Effect of sample pooling on the number of identified DMPs. Small filled triangles and box-and-whisker plot indicate distribution of differentially methylated positions (DMPs) that were called by comparing individual plants of generations 31 and 32 of mutation accumulation (MA) lines 30–39 and 30–49 with individual plants of 0-4-26 and 0-8-87, which represent generation 3 of two independent lines (16 comparisons in each group). The large unfilled triangles indicate the number of DMPs that were called when data from four lines of generations 31 and 32 were pooled in silico and compared against all four lines from generation 3. On average, a substantially lower number of DMPs is called with pooled data.
(EPS)

S11 Fig Characteristics of (differentially) methylated regions. (A) Histogram of the length of unified methylated regions (MRs), differentially and highly differentially methylated regions (DMRs and hDMRs). The red and green lines indicate mean and median length, respectively. For better visibility, regions larger than 2,000 bp were excluded from the representation. The minimum size of hDMRs was 50 bp. (B)-(D) are based on data of one HPG1 strain (LISET-036). (B) Number of unmethylated cytosines (Cs) in-between methylated CG sites (^m^CGs) within genes in dependence of whether these sequences are inside or outside of MRs. (C) Distances in bp between methylated CG sites within genes in dependence of whether these sequences are inside and outside MRs (minimal distance 2 bp). (D) Distances between methylated CG sites within body methylated (BM) genes identified with the method from ref. [48] (SOM: Validation of methylated regions) and within genes not identified as BM (minimal distance 2 bp). (E) Length distributions of DMRs that overlap and that do not overlap coding regions. Triangles show mean values.
(EPS)

S12 Fig Selecting parts of methylated regions to test for differential methylation. (A) Example illustrating the selection procedure of regions and pairwise strain comparisons to be tested for differential methylation. (*) For simplicity, the illustration uses a minimum number of covered sites of two reads per region (10 reads for the real data set). (1) We selected all possible regions where two strains presented different states of methylation (*reg1* to *reg5*) and applied filter criteria (a), (b) and (c). (2) If a region passed filters (a), (b) and (c) (in the example only *reg1* and *reg2*), criteria (a), (c) and (d) were checked for each pairwise comparison between strains on that region. Note, the selection of a region in (1) must not necessarily lead to a differential test between any two strains (e.g., *reg1*). Refer to the Material and Methods section for elaborate descriptions of criteria. (B) Assignment of strains to different groups based on differential methylation. Left: An exemplary differentially methylated region (DMR) represented as a graph: strains are represented as nodes, edges reflect a statistically significant test between two strains. Right: Finding the minimal number of sets, where no edge connects nodes from two different groups is known as the colouring vertex problem. In this example, the solution is two sets of strains (blue and white nodes). Strains without statistically significant tests (e.g., strain L) are grouped into the set of strains where the difference between the strain's and the group's mean methylation rate is minimal.
(EPS)

S13 Fig Methylation rate differences in regions of differential methylation. Histograms of the absolute mean methylation rate differences of differentially methylated regions (DMRs; grey) and highly differentially methylated regions (hDMRs; black) of all different sequence contexts.
(EPS)

S14 Fig Differential gene expression. (A) Hierarchical clustering of HPG1 accessions by expression of differentially expressed genes. (B) Differentially expressed genes per pairwise comparison. FPKM: fragments per kilobase per million mapped reads.
(EPS)

S15 Fig Differentially methylated regions (DMRs) and gene expression. Examples of DMRs (top panel) overlapping with a protein-coding gene (AT4G09360, left), a non-coding RNA (AT4G04223, middle) and a transposable element (AT1G62460, right). The expression of the corresponding locus is represented in the bottom panel. FPKM: fragments per kilobase per million mapped reads, 5^m^CG/5^m^CHG/5^m^CHH: methylated cytosine of respective context.
(TIF)

S16 Fig Distribution and annotation of single site epimutations in two populations. (A) Relative density of differentially methylated positions (DMPs) along the 5 Arabidopsis chromosomes according to their status of overlap between mutation accumulation (MA) and haplogroup-1 (HPG1) lineages. For each class and each chromosome, the window with the maximal density was set to 1. Sliding window; window size 100,000 bp; step size 10,000 bp. (B) Ratios between epimutation frequencies and sequencing depth along the 5 chromosomes for MA and HPG1 lines. Epimutation frequencies were determined as the number of DMPs per cytosine with at least threefold coverage per window. Coverage is represented as average coverage per window across all accessions of each population. Dashed lines mark the balanced coverage ratio of 1. Sliding window; window size 100,000; step size 10,000 bp. (C) Annotation of all cytosines (Cs), non-differentially methylated positions (N-DMPs) and DMPs according to their overlap between MA and HPG1 lineages. Cytosines were hierarchically assigned to CDS> intron> 5′ UTR> 3′ UTR> transposon> intergenic.
(EPS)

S17 Fig Overlap of MA and HPG1 DMPs according to MA generational distance. We computed differentially methylated positions (DMPs) between two randomly chosen mutation accumulation (MA) strains separated by specific numbers of generations and plotted the fraction of those DMPs shared with a randomly chosen haplogroup-1 (HPG1) strain. Each boxplot summarizes ten such random comparisons.
(EPS)

S18 Fig Overlap of MA and HPG1 DMRs per genomic feature and DMR sequence context. Differentially methylated regions (DMRs) were hierarchically assigned to CDS> intron> 5′ UTR> 3′ UTR> transposon> intergenic. CG-DMRs are differently methylated regions in the CG context only and C-DMRs in any other (additional) context(s).
(EPS)

S19 Fig Epigenetic variation in HPG1 lines and methylation-deficient mutants. (A, B) Fraction of sites that lost (A) or gained (B) methylation in mutant samples compared to two wild type (WT) Col-0 samples [43], from all methylated positions in WT, for each subset of these sites according to the status in the haplogroup-1 (HPG1) and mutation accumulation (MA) population (DMP: differentially methylated position). The set of all methylated positions in the wild type samples was restricted to sites covered in both HPG1 and MA lines as well as in the mutant sample (see Methods). (C, D) Fraction of sites that lost (C) or gained (D) methylation in mutant samples compared to WT samples, from all methylated positions in WT. Plotted are the fractions from all covered sites in all samples and from sites covered by DMRs within the haplogroup-1 lines that overlap regions of the genome with methylation occurring only in the CG context (^m^CG) or in any additional or alternative context(s) (^m^C).
(EPS)

S20 Fig Heritability of hDMRs by sequence context and status in MA lines. Distributions of heritability values of highly differentially methylated regions (hDMRs) according to significant sequence context and methylation status of overlapping regions in the mutation accumulation (MA) lines.
(EPS)

S21 Fig Hierarchical clustering by differentially and invariantly methylated positions and regions. DMPs: differentially methylated positions, DMRs: differentially methylated regions, N-DMPs: not differentially methylated positions, N-DMRs: not differentially methylated regions.
(EPS)

S22 Fig Analysis of hDMRs unique to LI-SET-036. (A) The number of strains sharing the same methylation status for highly differentially methylated regions (hDMRs) found in each strain is plotted (determined by the strain grouping procedure; see Methods). (B) Stacked bar plots showing the distributions of sequence contexts (bottom) and overlapping genomic features (top three plots) for hDMRs unique to each strain. ‘CG only’ exclusively considers CG-hDMRs whereas ‘CHG’ and ‘CHH’ might additionally include hDMRs of other contexts than CHG and CHH, respectively. The distribution across intergenic space, TEs and genes was similar for all strains. See section “Analysis of LI-SET-036 specific hDMRs” above for more details.
(EPS)

S1 Table Haplogroup-1 (HPG1) accessions used in this study.
(ODS)

S2 Table Summary statistics on genome sequencing.
(ODS)

S3 Table Common SNPs and SVs.
(TXT)

S4 Table Segregating SNPs and SVs.
(TXT)

S5 Table Summary statistics on methylome sequencing.
(ODS)

S6 Table Methylated regions (MRs).
(TXT)

S7 Table Differentially methylated regions (DMRs).
(TXT)

S8 Table Highly differentially methylated regions (hDMRs).
(TXT)

S9 Table Summary statistics on transcriptome sequencing.
(ODS)

S10 Table Differentially expressed (DE) genes identified in pairwise comparisons between HPG1 accessions. All DE genes (q-value <0.05) between any two accessions are listed. If more than one gene name appears in column 1, the read counts could not be assigned to one gene in particular and/or a fused transcript was suggested by the read data.
(ODS)

S11 Table Statistics of overlapping DE genes and hDMRs.
(ODS)

S12 Table Overlap of SVs with MRs in HPG1 and Col-0 in different genomic features.
(ODS)

S13 Table Scoring matrices for SNP calling and assessing cytosine site statistics (for bisulfite sequencing).
(ODS)

S1 File Command lines for software packages used in this study.
(PDF)
