# Supplementary material for: Century-scale Methylome Stability in a Recently Diverged Arabidopsis thaliana Lineage
Source: PLoS Genet. 2015 Jan 8;11(1):e1004920. doi: 10.1371/journal.pgen.1004920 (PMC4287485; doi:10.1371/journal.pgen.1004920)
Supplement: S1 File — Command lines for software packages used in this study. (PDF) [file pgen.1004920.s036.pdf]

Supplementary Online Material

Century-scale methylome stability in a recently diverged *Arabidopsis thaliana* lineage

Jörg Hagmann, Claude Becker, Jonas Müller, Oliver Stegle, Rhonda C. Meyer, George Wang, Korbinian Schneeberger, Joffrey Fitz, Thomas Altmann, Joy Bergelson, Karsten Borgwardt, Detlef Weigel

Genome analysis of HPG1 individuals

To answer how heritable epigenetic differences are affected by long-term exposure to fluctuating and diverse environmental conditions, we selected 13 haplogroup-1 (HPG1) individuals from seven locations in the Eastern Lake Michigan area, from one location in Western Illinois, and one location on Long Island; the median distance between sites was 155 km. The set consisted of pairs of accessions from each of four sites, and single individuals from the other five sites (Figure 1a, Table S1).

We assessed genetic divergence among the 13 HPG1 lines by Illumina paired-end sequencing (Table S2). We identified an initial set of common single-nucleotide polymorphisms (SNPs), small-scale indels and structural variants (collectively referred to as SVs henceforth) by read alignment to the Col-0 reference genome. We iteratively built an HPG1 pseudo-reference genome through integration of the common variants into the Col-0 genome, re-alignment of HPG1 reads to this new reference and re-calling of SNPs and SVs (see Method section; Figure S3a, c), following the rationale of Gan and colleagues[1]. After two iterations, the number of common variants had increased by 12% and the number of reads that could not be mapped had decreased by a third (Figure S3b). This ultimately identified 670,979 common SNPs and 170,998 SVs compared to the Col-0 reference (Table S3). Considering the corresponding nucleotides in the close relative *A. lyrata* as the ancestral states [2], a little bit more than half of the SNPs at positions alignable to the *A. lyrata* genome were classified as derived in the HPG1 population, close to what would be expected for a comparison of an arbitrary set of accessions (i.e., the HPG1 ancestor and Col-0) (Table S3). We additionally called variants in each of the 13 individuals based on the HPG1 pseudo-reference genome. Compared to the common variants, a much smaller number, 1,354 SNPs and 521 SVs, segregated in the HPG1 population (Table S4), confirming that the 13 strains were indeed closely related. As for common variants, segregating variants were fairly equally distributed across all ten chromosome arms (Figure S4). Eighteen percent of both segregating and common SNPs mapped to genes, and 30% of segregating and 35% of common SNPs to non-transposable element intergenic regions (Figure S5; Table S4). This was similar to SNPs in other natural accessions [3], indicating that HPG1 is representative of natural accessions of *A. thaliana*. On average, two HPG1 accessions differed by 294 SNPs.

Estimating DMP accumulation rates

We had previously sequenced the genomes of only five of the 12 MA lines for which we had reported [4,5] DMPs ’. We therefore generated additional genome sequence data for all ten MA lines in generation 31, counting from the founder plant of the population, as well as from the two lines in generation 3 [4]. To increase the number of data points in the low range of genetic differences, we inferred the number of SNPs between siblings (which had been included in the previous MA methylome analyses[4]) from the mutation rates determined by Ossowski and colleagues on the same lines. The greater variance in DMP rates and the more rapid initial increase in DMPs in the MA lines in Figure 2b is presumed to be due to the methylome data having come from individual plants, instead of from pools of individuals as for the HPG1 lines. By pooling strains, low frequency epimutations are diluted and less likely to be detected. This assumption is corroborated by the fact that we see only 46 DMPs between replicates of HPG1 pools compared to about 1,300 in replicates of the MA lines. To further investigate this, we compared the number of DMPs after *in silico* pooling of individuals. We first combined data from two siblings of MA line 30-39 or 30-49 in generation 31 with two siblings of their generation 32 offspring. We then calculated DMPs in comparison of pooled data from two times two individuals from two different generation 31 MA lines. We compared the results with those from individual comparisons of all 16 pairwise comparisons between the four early- and four late-generation individuals. The number of DMPs distinguishing pools was at the lower end of the DMP distribution from the individual comparisons

(Figure S10). Hence, it is likely that we underestimate the epimutation rate of the HPG1 accessions (Figure 2b). Moreover, we assumed the same genetic mutation rate in the two populations. A potentially faster genetic mutation rate in the wild would result in a steeper slope of the HPG1 curve, if plotted against the number of generations. Finally, the initial increase of the HPG1 epimutation rate is based on only few comparisons between strains from the same sampling site, which might not be sufficient for an accurate estimate. Since we assumed that the reported effects had a limited impact only (i.e., that the number of DMPs after pooling was still in the range of observed DMP numbers for individual comparisons), we conclude that it is unlikely that the epimutation rate in the wild is higher than in the greenhouse.

Validation of methylated regions

For validation of our HMM-based methylated region detection method, we compared data generated from Col-0 (see below) to data from methylated-DNA immunoprecipitation followed by sequencing (MeDIP-seq; Vincent Colot and co-workers, pers. communication). Of the genome space enriched in MeDIP-seq, 89% was classified as MR by our HMM approach.

To also evaluate whether the identified MRs sufficiently capture methylated sites within gene bodies consisting almost exclusively of CG sites, we tested how many MRs fall into gene body methylated genes as defined previously [6]. We re-implemented their method and called between 4,330 and 4,626 gene body methylated (BM) genes and between 14,998 and 15,489 unmethylated (UM) genes for the HPG1 strains. These figures are similar to the 4,361 BM and 15,753 UM genes reported in ref. [6] for Col-0. A quarter of the BM genes identified in this study and in ref. [6] did not overlap, which may be due to genetic differences and/or different sequencing depths and analysis pipelines. MRs in this work overlap with 58% of the HPG1 BM genes. This compares with an overlap of MeDIP domains of Col-0 (Colot lab, see above) of only 42% with Col-0 BM genes. Moreover, the concepts of our approach and that in ref. [6] differ considerably: by modeling the density of methylated sites within a gene with a binomial distribution allowing only little variance, genes with slightly increased density of methylated sites compared to the global average are quickly classified as BM in the method of ref. [6]. Such sites can still be located far apart from each other. In contrast, MRs are called by our HMM-based method only when there is a locally restricted, dense region of methylated sites. BM genes that are covered by MRs have a higher density of methylated sites compared to BM genes without overlapping MRs (Figure S11d).

Differentially methylated regions

To identify DMRs, we performed pairwise comparisons of overlapping MRs or parts thereof that were classified either (i) as in high methylation state in both accessions of a pair, or (ii) as high methylation state fragments in one and low methylation state in the other accession. For each DMR we then assigned all accessions to groups, based on their significant methylation differences (Figure S12, Method section). We expected to find fewer DMRs in regions that had a high methylation state in both tested accessions. In agreement, only 0.4% of those tested fragments (31,531 out of 7,355,716) were significantly differentially methylated. In contrast, we identified as differentially methylated 4.4% (107,988 out of 2,450,278) of fragments where the tested accessions had been assigned to contrasting methylation states.

In contrast to previously used methods for the analysis of whole-genome bisulfite sequencing data from plants, our HMM for the detection of MRs does not require information about methylation differences at the single-site level. By

first identifying blocks of methylation, our approach limits the number of tested regions to the methylated space of the genome, thereby reducing the multiple testing problem and the requirement for arbitrary filters. Importantly, limiting DMR detection to HMM-identified MRs revealed that the location of DMRs in the genome follows the overall distribution of methylation. Most of these DMRs thus overlap with TEs and intergenic regions, which is in contrast to previously published DMRs relying on user-defined criteria including arbitrary sliding windows or distance-based clustering of differentially methylated positions [4,7-16]. The HMM analysis also revealed that non-CG methylation is almost exclusively organized in regions of contiguous DNA methylation. We suggest such an approach to identifying MRs be applied to bisulfite sequencing data in future studies.

Analysis of LISET-036 specific hDMRs

Strain LISET-036 was the most different when strains were clustered by CHG-DMPs, CHH-DMPs and DMRs. Since CHG and CHH-DMPs constituted only a minor fraction of all DMPs (~3%), we focused on hDMRs private to the HPG1 strains to investigate the possible basis of LISET-036 being an outlier. While LISET-036 had the most private hDMRs among all accessions (Figure S22a), their spectrum in terms of context and overlap with genomic features did not deviate from that of the other strains (Figure S22b). 44 LISET-036 private hDMRs overlapped with genes and 30 overlapped with the gene-adjacent regions, defined as 1,000 bp upstream or downstream of genes. The only GO term for which these 74 genes were enriched was "intrinsic to membrane" (p-value 0.01). However, there were no overlapping differentially expressed genes. Taken together, there is little evidence for a pronounced phenotypic effect of the LISET-036-specific epivariants.

Differential gene expression and epigenetic variation

We performed RNA-seq (Table S9) on rosette leaves of all 13 strains and identified 251 differentially expressed (DE) genes across all possible pairwise comparisons (Table S10). A majority of these genes were identified as DE in more than one comparison. Gene Ontology (GO) analysis identified several defense-related GO terms to be over-represented ($p << 0.001$); which may be linked to these genes evolving fast[17]. As could be expected from the small numbers of DE genes, clustering of accessions based on expression of all genes revealed no structure reflecting genetic distance or geographical origin. When we limited the clustering to DE genes, however, accessions originating from the same geographical location, except Yng-4 and Yng-53, clustered together (Figure S14a). A similar observation could be made when counting the number of DE genes per comparison: while accessions from the same site generally showed no or only few DE genes, comparison of accessions from different sites revealed up to 149 differences. The two Yng strains accounted for most of the DE genes identified in pairwise comparisons (Figure S14b). Although we identified some loci where changes in contiguous stretches of DNA methylation correlated with alterations in transcriptional activity (Table S11), we did not observe a general relationship between these two features, suggesting either that transcriptional differences in the haplogroup-1 (HPG1) population is mostly due to DNA mutations, or due to epigenetic changes independent of DNA methylation.

Supplementary References

1. Gan X, Stegle O, Behr J, Steffen JG, Drewe P, et al. (2011) Multiple reference genomes and transcriptomes for *Arabidopsis thaliana*. Nature 477: 419-423.

2. Hu TT, Pattyn P, Bakker EG, Cao J, Cheng JF, et al. (2011) The *Arabidopsis lyrata* genome sequence and the basis of rapid genome size change. Nat Genet 43: 476-481.

3. Cao J, Schneeberger K, Ossowski S, Gunther T, Bender S, et al. (2011) Whole-genome sequencing of multiple *Arabidopsis thaliana* populations. Nat Genet 43: 956-963.

4. Becker C, Hagmann J, Müller J, Koenig D, Stegle O, et al. (2011) Spontaneous epigenetic variation in the *Arabidopsis thaliana* methylome. Nature 480: 245-249.

5. Ossowski S, Schneeberger K, Lucas-Lledo JI, Warthmann N, Clark RM, et al. (2010) The rate and molecular spectrum of spontaneous mutations in *Arabidopsis thaliana*. Science 327: 92-94.

6. Takuno S, Gaut BS (2012) Body-methylated genes in *Arabidopsis thaliana* are functionally important and evolve slowly. Mol Biol Evol 29: 219-227.

7. Schmitz RJ, Schultz MD, Lewsey MG, O'Malley RC, Urich MA, et al. (2011) Transgenerational epigenetic instability is a source of novel methylation variants. Science 334: 369-373.

8. Dowen RH, Pelizzola M, Schmitz RJ, Lister R, Dowen JM, et al. (2012) Widespread dynamic DNA methylation in response to biotic stress. Proc Natl Acad Sci USA 109: E2183-2191.

9. Schmitz RJ, Schultz MD, Urich MA, Nery JR, Pelizzola M, et al. (2013) Patterns of population epigenomic diversity. Nature 495: 193-198.

10. Schmitz RJ, He Y, Valdes-Lopez O, Khan SM, Joshi T, et al. (2013) Epigenome-wide inheritance of cytosine methylation variants in a recombinant inbred population. Genome Res.

11. Qian W, Miki D, Zhang H, Liu Y, Zhang X, et al. (2012) A histone acetyltransferase regulates active DNA demethylation in Arabidopsis. Science 336: 1445-1448.

12. Calarco JP, Borges F, Donoghue MT, Van Ex F, Jullien PE, et al. (2012) Reprogramming of DNA methylation in pollen guides epigenetic inheritance via small RNA. Cell 151: 194-205.

13. Ausin I, Greenberg MV, Simanshu DK, Hale CJ, Vashisht AA, et al. (2012) INVOLVED IN DE NOVO 2-containing complex involved in RNA-directed DNA methylation in *Arabidopsis*. Proc Natl Acad Sci USA 109: 8374-8381.

14. Stroud H, Greenberg MV, Feng S, Bernatavichute YV, Jacobsen SE (2013) Comprehensive analysis of silencing mutants reveals complex regulation of the Arabidopsis methylome. Cell 152: 352-364.

15. Regulski M, Lu Z, Kendall J, Donoghue MT, Reinders J, et al. (2013) The maize methylome influences mRNA splice sites and reveals widespread paramutation-like switches guided by small RNA. Genome Res 23: 1651-1662.

16. Stroud H, Ding B, Simon SA, Feng S, Bellizzi M, et al. (2013) Plants regenerated from tissue culture contain stable epigenome changes in rice. Elife 2: e00354.

17. Jones JD, Dangl JL (2006) The plant immune system. Nature 444: 323-329.
